# T-Cell Receptor Repertoire Characteristics Associated with Prognostic Significance in High-Grade Serous Ovarian Carcinoma

**DOI:** 10.3390/genes14040785

**Published:** 2023-03-24

**Authors:** Ju-Won Kim, Sewha Kim, So-Yun Yang, Je-Gun Joung, Sohyun Hwang

**Affiliations:** 1 Department of Biomedical Science, College of Life Science, CHA University, Seongnam 13488, Republic of Korea; seven1009@naver.com (J.-W.K.);; 2 Department of Pathology, CHA Bundang Medical Center, CHA University School of Medicine, Seongnam 13496, Republic of Korea; 3 Department of Pathology, Korea Medical Institute, Seoul 03173, Republic of Korea; 4 Institute for Biomedical Informatics, CHA University School of Medicine, Seongnam 13488, Republic of Korea; 5 CHA Future Medicine Research Institute, CHA Bundang Medical Center, Seongnam 13488, Republic of Korea

**Keywords:** ovarian cancer, high-grade serous carcinoma, t-cell receptor, immune pathway

## Abstract

High-grade serous ovarian carcinoma (HGSOC) is a fatal gynecological malignancy. Somatic recombination occurring during T-cell receptor (TCR) development results in TCR diversity, and the TCR repertoire, thus produced, is associated with immune response. This study analyzed the difference in the TCR repertoire and their prognostic significance in 51 patients with HGSOC. The patient’s clinical characteristics, gene expression pattern, TCR clonotypes, and degree of tumor-infiltrating leukocytes (TILs) were analyzed, and the patients were divided into groups depending on their recurrence pattern, tumor-infiltrating leukocyte (TIL) score, and homologous recombinant repair pathway deficiency (HRD)-associated mutations. The TCR repertoire was low in patients with recurrence and showed the expansion of eight TCR segments. Interestingly, a few genes correlated with the TCRs also showed a difference in expression according to the prognosis. Among them, seven genes were related to immune responses and *KIAA1199* was up-regulated in ovarian cancer. Our study shows that the differences in the TCR repertoire in patients with ovarian cancer and their associated immune pathways could affect the prognosis of HGSOC.

## 1. Introduction

Despite its rare incidence, ovarian cancer remains a fatal gynecological malignancy. Most ovarian cancers are epithelial in origin, known as epithelial ovarian cancer, and rank eighth in women’s cancer-related deaths [1,2]. The most common histological subtype of epithelial ovarian cancer is high-grade serous ovarian cancer (HGSOC), accounting for 60% to 80% of all cases [3]. Women with a family history of ovarian cancer have a significantly higher risk of developing ovarian cancer than women without a similar family history [4]. A reproductive mutation in the tumor suppressor genes *BRCA1* and *BRCA2* contributes to an increased risk of developing ovarian cancer within a family [5]. Homologous recombination repair (HRR) pathway deficiency (HRD) contributes greatly to the pathogenesis of HGSOC and is characterized by increased chromosomal instability [6]. HRD is caused by germline or somatic *BRCA1/2* mutations, hypermethylation at the *BRCA1/2* gene promoter, and other genetic abnormalities in the HRR pathway [6,7]. Interestingly, patients with HRD show a good prognosis for ovarian cancer, as platinum-containing drugs can cause irreversible DNA damage, leading to the apoptosis of cancer cells [8]. However, the likelihood of recurrence and a poor prognosis remains challenging in treating patients with ovarian cancer [7].

T-cell-mediated antigen recognition and inflammatory response relies on the interaction between the antigen-major histocompatibility complex (MHC) molecules and T-cell receptors (TCRs). TCRs are heterogeneous dimers, consisting of a combination of α and β chains (αβ TCR) expressed in most T cells or γδ chains (γδ TCRs) expressed in peripheral blood (1–5%) T cells [9]. Structurally, the TCR β chain consists of variable (V), diversity (D), joining (J), and constant regions (C). In contrast, the TCR α chain consists only of the regions V, J, and C [10]. TCRs are generated by the V(D)J recombination, a process involving a random combination of V, J, and in some cases, D segments. This process produces various TCRs depending on the segments that are recombined and the addition or deletion of various nucleotides. The TCR repertoire refers to the sum of TCRs in individuals; V(D)J recombination increases the TCR diversity and TCR repertoire [9,11]. Moreover, TCR repertoire diversity increases immunity levels in various diseases [12,13]. 

Tumor-infiltrating lymphocytes (TILs) are infiltrated immune cells in tumors and include T cells, B cells, and natural killer (NK) cells. [14]. Stromal TILs, such as B and T cells, act as major immune cells in the tumor microenvironment (TME), secreting cytokines and activating the immune response, and have been recognized to directly or indirectly affect patient survival [15,16]. In particular, previous studies have shown that TIL-related cytokines, such as INF-γ, IL-2, IL-10, and TGF-β, are associated with TIL inhibition and tumor overgrowth in ovarian cancers. Furthermore, these cytokines have been investigated as potential therapeutic targets [17]. In clinical practice, TILs have been suggested as a biomarker of improved prognosis and overall survival and are associated with ovarian and breast cancer [15]. In particular, stromal TILs are associated with a better prognosis in HGSOC [18].

We, therefore, hypothesized that the number or diversity of TCRs might be related to the immune response of ovarian cancer. We attempted to identify specific TCR segments that differ in expression depending on the prognosis groups and identify their associated genes and pathways.

## 2. Materials and Methods

### 2.1. RNA Sequencing Data of HGSOC Patients

We downloaded 51 raw RNA FASTQ files (SRP303861) from the Sequence Read Archive (SRA) database and 51 raw RNA count data and clinical information (GSE165808) from the Gene Expression Omnibus (GEO) database. All 51 patients had undergone a radical hysterectomy with salpingo-oophorectomy and platinum-based chemotherapy for ovarian carcinomas. RNA was extracted from the individual fresh snap-frozen tissue samples, RNA sequencing libraries were prepared using the Illumina TruSeq standard mRNA Prep kit, and FASTQ files were generated using the HiSeq 2500 sequencing system (Illumina, San Diego, CA, USA) [19]. We also downloaded and analyzed clinical data and the RNA raw expression data of 396 high-grade serous ovarian cancer patients from TCGA in cBioPortal (https://www.cbioportal.org) (accessed on 28 February 2023) [6].

### 2.2. TCR Analysis

In the pretreatment process, only the V, D, J, and C regions of the T-cell receptor were aligned and assembled using MiXCR (1.8.1 version) to obtain TCR clonotypes of each patient from the raw RNA FASTQ data [20]. On aligning, it was set as the type of *Homo sapiens* embedded in the MiXCR. The obtained data were analyzed using VDJtools (1.2.1 version) [21] and the immunarch R package (https://immunarch.com/) (accessed on 19 December 2022) to analyze the number of TCR clonotypes, repertoire diversity, and clonality. The TCR repertoire diversity was estimated using the Chao1 and D50 diversity index. Chao1 is the nonparametric asymptotic estimator of species richness (number of species in a population), and the D50 diversity index is a recently developed immune diversity estimate [19].

### 2.3. RNA Expression Analysis

To identify differentially expressed (DE) TCR gene segments, we conducted a two- sample t-test, performed differently depending on whether the variance was the same or different. As a result of the t-test, only those gene segments with a *p*-value < 0.05 were extracted, because there were only two with a false discovery rates (FDR) < 0.05 for the DE TCRs.

Differentially expressed genes (DEG) were analyzed with raw RNA count data using the edgeR R package [22]. In this process, we applied the generalized linear model approach using the quasi-likelihood F-test method. The quasi-likelihood method provides stricter error rate control by accounting for the uncertainty in the dispersion estimation in bulk RNA-sequencing data. When obtaining DEG, we used FDR to correct multiple comparisons. As a result of the DEG, only those with an FDR of 0.05 or less were extracted.

The gene ontology biological process and KEGG pathway analyses were conducted using g:Profiler (https://biit.cs.ut.ee/gprofiler) (accessed on 12 January 2023).

### 2.4. TIL Analysis

The degree of TILs was interpreted using the most representative whole tissue sections of ovarian carcinomas. The interpretation followed the recommendation of the International Immuno-Oncology Biomarkers Working Group [23]. TILs were defined as the mononuclear cells that infiltrated the stroma between tumor cells and were evaluated within the invasive border. The TIL percentage (%) score was the proportion of the stromal area occupied by mononuclear cells over the total stromal area.

### 2.5. Survival Analysis

Disease-free survival (DFS) analysis was analyzed with the raw RNA count data using the survival and survminer R packages. The analysis was performed by dividing patients into ‘High’ and ‘Low’ groups based on the median value of the gene expression. 

### 2.6. Statistical Analysis

Volcano plots were generated using the ggplot2 R package, and the difference in the expression of TCR gene segments between groups was analyzed by the t-test using the stats R package. A heatmap of the TCR raw count data of the VDJtools results was drawn using the Complex Heatmap R package [24].

## 3. Results

### 3.1. Clinical Characteristics of Patients with HGSOC

In our previous study, we generated and investigated the whole transcriptome of 51 patients with HGSOC to identify the key prognostic markers, *USP19* and *RPL23,* for advanced-stage HGSOC [25]. In this study, we investigated the same 51 patients with HGSOC, and the characteristics of these patients are described in Table 1. The average age of the patients was 55 years (range, 36–77 years), with 80.4% and 9.8% belonging to stage 3 and stage 4, respectively.

In this study, first, the patients were divided into two groups, no-recurrence and recurrence groups based on recurrence, except for three patients who were lost to follow up. The number of patients in the no-recurrence and recurrence groups was 10, and 38, respectively, and the study was conducted in these two groups.

Second, the patients were divided based on their TIL percentage (%) scores into three groups—those with a TIL score of less than 20, between 20 and 40, and greater than 40—and included 21, 16, and 10 patients, respectively. Four patients had no information on TIL scores.

Lastly, we divided the patients into three groups based on the presence of mutations in the HRD genes (Table 1). Among the twenty-two patients with HRD mutations (HRD-positive), eighteen had the *BRCA1/2* mutation [26], and four had mutations in other HRD genes, such as *ATR*, *MSH2*, *MSH6*, *RAD50*, and *FANCA*. Twenty-seven patients without HRD mutations were classified into the HRD-negative group. Two patients had no information regarding HRD mutations.

### 3.2. The Expression Pattern of TCR Repertoire Genes in Patients with HGSOCs

**Figure 1:**
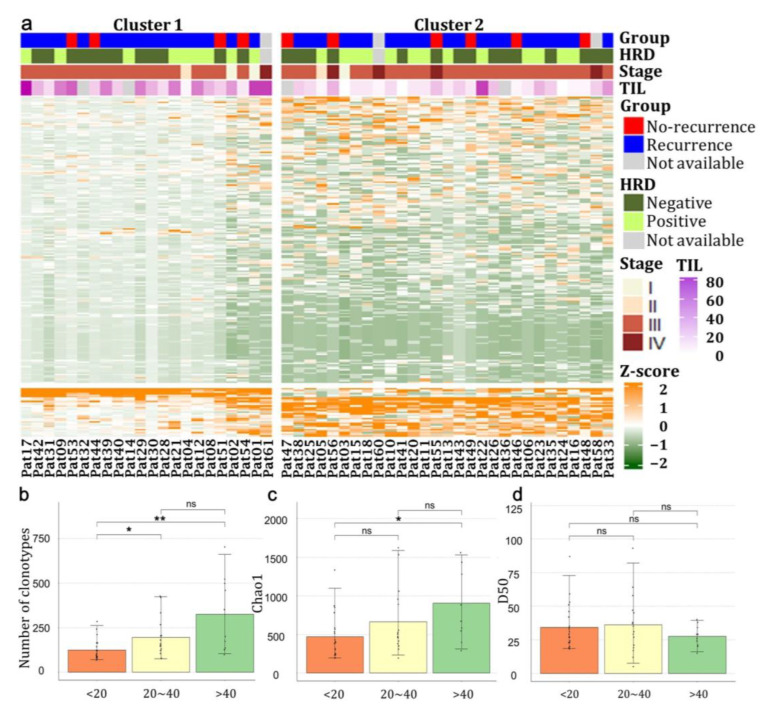
Distribution of T-cell receptor counts was associated with TIL scores. (**a**) Expression of TCR segments was standardized in each patient (the column of the heatmap). Recurrence, HRD mutation, stage, and TIL scores were annotated for each patient; (**b**) The counts of unique TCR clonotypes; (**c**,**d**) The richness and diversity of TCRs were estimated using Chao1 and the D50 diversity index, respectively, in patients divided based on their TIL percentage (%) scores. (**b**–**d**) Non-significant *p*-values are indicated as ns, significant *p*-values < 0.05 are indicated as *, and significant *p*-values < 0.01 are indicated as **.

In the heatmap shown in Figure 1a, the expression of TCRs in patients was standardized based on each patient. Both patients and TCR segments were clustered hierarchically and divided into two clusters using k-means clustering. The TIL percentage (%) scores of the patients showed a distinct association with the two clusters (Cluster 1 and Cluster 2) (*p* = 4.0 × 10^−4^). In Cluster 1, the TIL scores were higher, and TCRs seemed to be variously distributed. Conversely, in Cluster 2, the TIL scores tended to be lower, and specific TCRs were highly expressed, implying a lower diversity of the expressed TCRs.

To confirm, we divided the patients into three groups (TIL < 20, 20 ≤ TIL ≤ 40, and TIL > 40) based on their TIL scores and analyzed the TCR expression pattern in each group (Figure 1b–d). The number of TCR clonotypes increased as the TIL scores increased. In particular, the number of clonotypes between TIL < 20 and TIL > 40 (*p* = 0.004), and between TIL < 20 and 20 ≤ TIL ≤ 40 (*p* = 0.04) were significantly different (Figure 1b). We also analyzed whether TCR richness and diversity differed between the TIL groups using Chao1 and the D50 diversity index. Chao1 was significantly higher in TIL > 40 than in TIL < 20 (*p* = 0.02; Figure 1c). However, no significant differences were observed in the D50 diversity index (Figure 1d).

### 3.3. The Expression Pattern of TCR Repertoire Genes between Recurrence and No-Recurrence Groups

**Figure 2:**
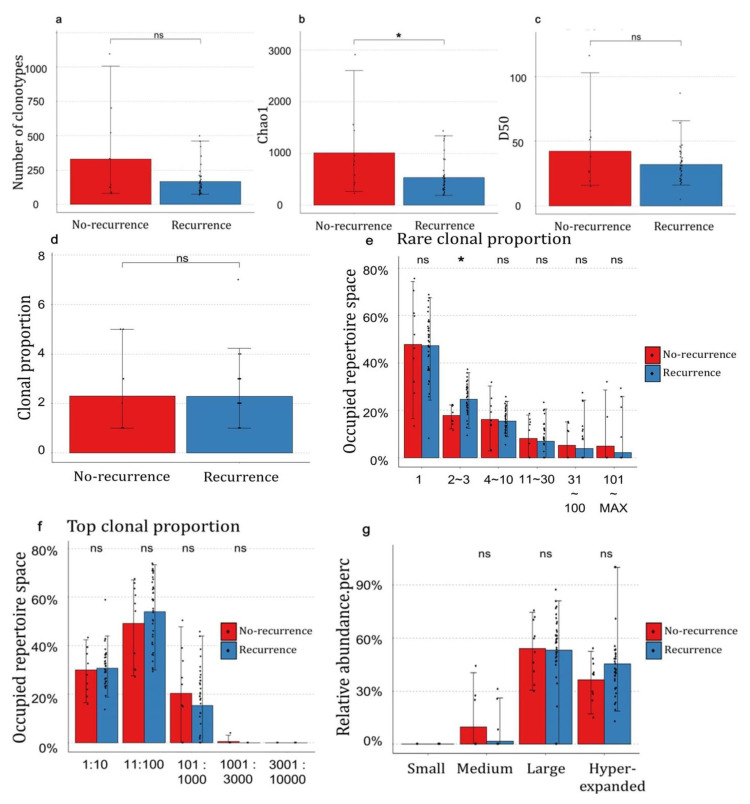
Expression patterns of TCR repertoire genes according to the patient’s prognosis. (**a**) The counts of unique TCR clonotypes; (**b**,**c**) The richness and diversity of TCR between the recurrence and no-recurrence groups estimated using Chao1 and the D50 diversity index; (**d**–**g**) The clonality of TCR segments between the recurrence and no-recurrence groups; (**d**) The numbers of clonotypes occupying 12% of the TCR repertoire; (**e**) The proportion of rare clonal clonotypes with specific counts; (**f**) The proportion of top clonal clonotypes with specific indices; (**g**) The relative abundance of the small, medium, large, and hyperexpanded clonotypes. (**a**–**g**) Non-significant *p*-values are indicated as ns, and *p*-values < 0.05 are indicated as *.

To understand the relationship between TCRs and prognosis, we divided the patients into two groups, recurrence and no-recurrence, and analyzed the number of TCR clonotypes, TCR richness, and TCR diversity between the groups (Figure 2a–c). The number of TCR clonotypes tended to be lower in the recurrence group, but the difference was not statistically significant (Figure 2a). TCR richness and diversity were measured using Chao1 and the D50 diversity index. The Chao1 result showed a significant difference between the groups (*p* = 0.03), suggesting lower TCR richness in the recurrence group (Figure 2b). The D50 diversity index showed the same trend as that of Chao1, but the difference was insignificant (Figure 2c). Our results indicate a difference in the richness of the TCR clonotypes and a reduced TCR repertoire diversity in the recurrence group.

### 3.4. The Distribution of Clonotypes Occupying TCR Repertoires between Recurrence and No-Recurrence Groups

Next, we studied whether the occupied proportions of the TCR repertoire between the recurrence and no-recurrence groups were different. The number of clonotypes occupying 12% of the repertoire showed no significant difference between the two groups (Figure 2d). For the proportion of rare clonal clonotypes, the *x*-axis represents the counts of the clonotypes, and a lower x value indicates a rare clonotype (Figure 2e). In most clonotype counts, there was no significant difference between the two groups, but in the count of clonotypes = 2~3, the result was significantly different (*p* = 0.021). This means that the occupied repertoire space was higher in the recurrence group at a count of clonotype = 2~3 (Figure 2e). In addition to the proportion of rare clonotypes, we also investigated the proportion of the top clonotypes. The *x*-axis in the proportion of top clonal clonotypes represents the clonotype index (Figure 2f). A small clonotype index indicates an expanded clonotype, and a large index indicates a small clonotype group. No clonotype index showed a significant difference between the groups in the top clonal proportion (Figure 2f). 

Finally, the relative abundance of the TCR clonotypes was measured. The *x*-axis of Figure 2g represents the clonotype groups, which were divided based on the expression levels of the clonotypes. The criteria for each group were as follows; small: 0 < log (expression) ≤ 10^−4^, medium: (10^−4^ < log (expression) ≤ 10^−3^), large: 10^−3^ < log (expression) ≤ 0.01, and hyperexpanded: 0.01 < log (expression) ≤ 1. The relative abundance of the clonotype groups did not differ significantly between the recurrence and no-recurrence groups (Figure 2g).

### 3.5. Differentially Expressed T-Cell Receptor Gene Segments and Genes between Recurrence and No-Recurrence Groups

**Figure 3:**
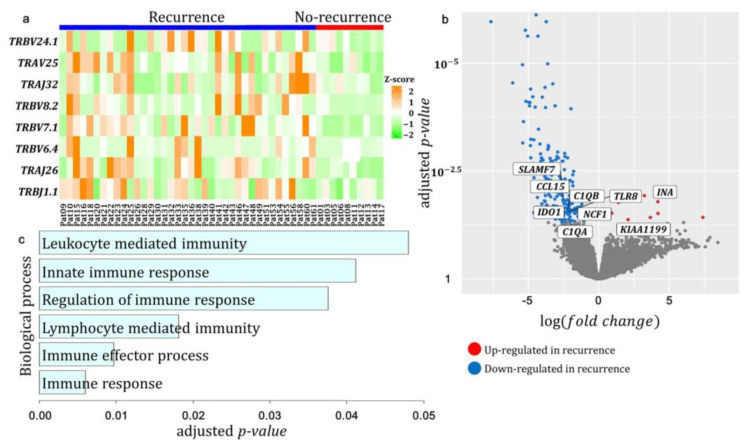
Genes correlated with specific TCRs and their enriched biological process. (**a**) Expression of eight TCR segment genes differentially expressed in recurred patients; (**b**) A volcano plot for differentially expressed genes (DEGs) between the recurrence and no recurrence groups. Fold change was calculated by dividing the recurrence group’s average expression by that of no recurrence group; (**c**) GO biological process terms that were significantly enriched in both DEGs and genes having negative correlation with those eight TCRs.

We identified eight differentially expressed TCR gene segments between the recurrence and no-recurrence groups, using two group t-tests with *p* < 0.05: *TRBV24.1* (*p* = 3.0 × 10^−2^), *TRAV25* (*p* = 8.0 × 10^−4^), *TRAJ32* (*p* = 3.0 × 10^−2^), *TRBV8.2* (*p* = 1.0 × 10^−3^), *TRBV7.1* (*p* = 4.5 × 10^−6^), *TRBV6.4* (*p* = 2.0 × 10^−3^), *TRAJ26* (*p* = 8.7 × 10^−5^), and *TRBJ1.1* (*p* = 1.0 × 10^−2^). All eight TCR segments were highly expressed in patients in the recurrence group (Figure 3a) and appeared to be involved in the hyperexpanded clonotypes in the recurrence groups.

We also performed a DEG analysis between the recurrence and no-recurrence groups using the whole transcriptome profile of each patient and identified 180 genes with adjusted *p* < 0.05 and |log (fold change)| > 1 (Figure 3b).

We then analyzed the correlation between the expression of 180 DEGs and eight DE TCR segments to identify genes associated with both TCR segments and patients in the recurrence. 

Among the 180 DEGs, 14 genes significantly correlated with the eight TCR segments (*p* < 0.01), with three positively correlated and 11 negatively correlated with the TCR segments. As shown in Figure 3c, many immune GO terms were significantly enriched in the 11 genes negatively correlated with TCR segments; immune response (*p* = 6.0 × 10^−3^), immune effector process (*p* = 9.0 × 10^−3^), lymphocyte mediated immunity (*p* = 1.0 × 10^−2^), regulation of immune response (*p* = 3.0 × 10^−2^), innate immune response (*p* = 4.0 × 10^−2^), and leukocyte mediated immunity (*p* = 4.0 × 10^−2^). These results showed that patients with no-recurrence demonstrated an increased immune response; however, in patients with recurrence, an increase in the expansion of the eight TCR segments limited diversity in the TCR repository, affecting their immune response.

The volcano plot in Figure 3b also shows each gene’s statistical significance and fold change. Two genes, *KIAA1199* and *INA*, were significantly increased in patients with recurrence than in patients with no-recurrence, and seven genes, *IDO1*, *SLAMF7*, *CCL15*, *C1QB*, *TLR8*, *NCF1*, and *C1QA,* were significantly decreased in patients with recurrence.

### 3.6. Prognosis of Differentially Expressed Genes in Patients with HGSC

We investigated whether the nine genes (*KIAA1199, INA, IDO1*, *SLAMF7*, *CCL15*, *C1QB*, *TLR8*, *NCF1*, and *C1QA)* were associated with the prognosis of ovarian cancer patients. Among these genes, *KIAA1199*, *IDO1*, *NCF1*, and *SLAMF7* showed significant results in the survival analysis. The prognosis of patients differed significantly by the expression of *KIAA1199* (*p* = 4.7 × 10^−2^) in our 51 patients but not in TCGA HGSC data (*p* = 0.63). The three genes (*IDO1*, *NCF1*, and *SLAMF7*) were negatively correlated with the TCR segments; the expression of these genes were not significantly different in all 51 patients but were significantly different in TCGA HGSC data (*IDO1* (*p* = 4.9 × 10^−2^), *NCF1* (*p* = 2.2 × 10^−2^), and *SLAMF7* (*p* = 1.0 × 10^−4^); in Figure S1).

### 3.7. Differential Expression of TCR Repertoires between HRD Mutation-Positive and Negative Groups

In ovarian cancers, HRD is important for determining a patient’s treatment. Previous studies have reported that patients with HRD mutations showed a better prognosis than those without these mutations; further, HRD mutations are associated with increased immune cell infiltration [27,28]. Therefore, we attempted to investigate the relationship between HRD and the characteristics of TCRs. We investigated whether the distribution of the TCR repertories was different in patients with HRD. The patients were divided into HRD mutation-positive or mutation-negative groups, depending on the presence or absence of mutations in HRD genes. The TCR expression in the two groups is shown in Figure S2. The number of clonotypes was smaller in the HRD mutation-negative group than in the HRD mutation-positive group, but the difference was not significant (Figure S2a). Next, the TCR repertoire diversity between the two groups was measured using Chao1 and the D50 diversity index. The TCR repertoire diversity between the groups showed a similar tendency. The TCR repertoire diversity was lower in the HRD mutation-negative group (Figure S2b,c), but the difference was not statistically significant.

## 4. Discussion

This study investigated how the TCR repertoire differs among patients with HGSOC. Analyzing the heatmap of the TCR repertoire for all patients (Figure 1a) showed a difference in TCR expression among them. The patients in Cluster 2 showed a high expression of specific TCRs; however, most TCRs continued to have a lower expression. The results imply that although many expanded TCRs existed, the number of TCRs and the TCR repertoire diversity were low. The most significant differences between patients in Clusters 1 and 2 were observed in the TIL scores. Patients in Cluster 1 had relatively higher TIL scores than those in Cluster 2. These results agree with those of the previous studies, which reported a correlation between the TIL score and the TCR repertoire. Specifically, a diverse TCR and a high TIL score is associated with a better prognosis [29,30].

Our results suggest that the number of clonotypes and the diversity of the clonotype repertoire in patients with HGSOC would differ according to the TIL level. The number of TCR clonotypes and TCR richness were significantly increased in the higher TIL score group (Figure 1b–d). The results were consistent with previous studies showing that high TIL scores are associated with increased TCR repertoires and play a more positive role in immune responses [29,30]. In other words, the presence of diverse TCRs would boost the immune response, resulting in high TIL levels.

We also determined whether the TCR repertoire differed according to patient prognosis. We observed that the number of clonotypes and TCR richness tended to be high in patients with no recurrence. In recent studies, patients with ovarian cancer with fewer TCR clonotypes have shown poor survival and prognosis [27]. Our results showed a similar tendency with patients with recurrence showing a lower diversity in the TCR repertoire. The diversity of the TCRs seemed to be correlated with the prognosis of patients, and the higher the diversity of the TCR repertoire, the better the prognosis.

We analyzed the TCR segments to determine which TCR genes showed differences in expression between the recurrence vs. no-recurrence groups and found a significant difference in eight TCR genes, with high expression in the recurrence group. Moreover, these eight TCRs seemed to have expanded in the recurrence group, resulting in low TCR repertoire diversity. The expansion of TCRs and the lower expression of other TCRs suggested a decrease in the TCR repertoire, affecting the immune response and increasing the likelihood of recurrence in patients with HGSOC.

Next, we conducted the DEG and correlation analyses on the differentially expressed genes, according to the recurrence groups, and correlated them with eight expanded TCRs. Genes negatively correlated with the expanded TCRs were greatly related to immunological process and had a lower expression in the recurrence group. Our results suggest that expanded TCRs and the reduced expression of their associated immune genes may cause the recurrence of ovarian cancer by affecting the patient’s immune response. Conversely, *KIAA1199*, which is positively correlated with eight expanded TCRs, is widely known to be up-regulated in ovarian cancer, according to previous studies [31,32]. Consistently, we observed a higher *KIAA1199* expression in recurrent patients, with a positive correlation with these TCRs.

We also investigated the relationship of nine genes to prognosis in ovarian cancer patients. Among these nine genes, four genes were associated with the prognosis of patients. *KIAA1199* was associated with a poor prognosis, as it was highly expressed in recurrent patients. As expected, in our 51 patients, those with a higher expression of *KIAA1199* had a worse prognosis, and this finding was statistically significant (Figure S1a). However, in TCGA HGSC patients, this finding was not statistically significant (Figure S1b). Thus, more research would be needed to clarify these findings. The three genes (*IDO1*, *NCF1*, and *SLAMF7*) were expected to be associated with a good prognosis, because they were negatively correlated with the eight TCR segments and had a lower expression in recurrent patients. As expected, in TCGA HGSC patients, those with a higher expression of these three genes had a better prognosis, and this finding was statistically significant (Figure S1d,f,h). However, this finding was not significant in the DFS data of our patients (Figure S1c,e,g). Therefore, the confirmation of the association of these genes with a good prognosis in other independent ovarian cancer data is required.

Lastly, as previous studies have shown a good prognosis of ovarian cancer in patients with HRD mutations, we analyzed whether there was a difference in the TCR repertoire depending on the HRD mutation [27]. As shown in Figure S2, patients with HRD mutations showed a higher number of clonotypes and higher TCR repertoire diversity than patients without HRD mutations; however, this difference was not statistically significant.

Our study showed that changes in the TCR repertoire affect the prognosis of ovarian cancer; preventing these changes could be a promising therapeutic approach to improve the diagnosis and treatment of patients with ovarian cancer. Since the decrease in the expression of seven genes related to the immune response and the increase in expression of the *KIAA1199* gene were significant in patients with recurrence, it is necessary to conduct further research on the relationship between TCRs and genes to identify the effect of each gene on ovarian cancer. In addition, further studies are required to analyze the difference in the TCR repertoire depending on the HRD mutation in patients with HGSOC.

This study has several limitations. First, no significant results were observed in the number of TCRs and clonality between the recurrence and no-recurrence groups and in the number of TCRs and TCR repertoire diversity between the HRD groups and could be attributed to the small samples. As a large-scale ovarian cancer cohort, TCGA ovarian cancer data was also investigated. However, we could not conduct the same analysis on TCR data from TCGA ovarian cancer data [33], because TCGA clinical data do not provide information on patients without recurrence. Instead, we analyzed the OS and PFI data of TCGA ovarian cancer patients according to the TCR richness and Shannon scores in the TCGA ovarian cancer data. TCR richness and the Shannon diversity index were not significantly associated with the survival of TCGA ovarian cancer patients.

Second, we could not investigate how to combine the *USP19* and *RPL23* markers and the TCR repertoire to improve the prediction accuracy of patient prognosis. The prediction model, based on *USP19* and *RPL23* expression, could predict prognosis in the advanced stage, but the TCR repertoire was associated with the recurrence of patients regardless of stage. Therefore, constructing a prediction model by combining these two markers and the TCR repertoire was challenging. In the future, we plan to study the prediction of prognosis in ovarian cancer by combining these markers.

Lastly, findings on the three genes, *IDO1*, *NCF1*, and *SLAMF7*, were statistically significant in the DFS analysis of TCGA HGSC patients, but not in those of our patients (Figure S1). It is likely that the significance of the results was affected by the small number of patients, and it is necessary to conduct further research in another large independent cohort.

## 5. Conclusions

We analyzed the TCR repertoire through RNA sequencing data from 51 patients with HGSOC and identified eight expanded TCR segments in recurrent patients, affecting the TCR repertoire, according to prognosis. We selected the genes that correlated with these TCRs and had a prognostic significance, and were identified as genes involved in the immune response and associated with ovarian cancer. We believe that the TCR segments and the associated genes identified might help understand the immune response involved in ovarian cancer, improving the treatment strategies.

## Figures and Tables

**Table 1:** Clinical Characteristics of Patients with HGSOC.

Characteristic	Overall (*n* = 51)	%
Age (Years), Median (Range)	55 (36–77)	
Stage		
I	3	5.9
II	2	3.9
III	41	80.4
IV	5	9.8
Platinum sensitivity		
No-recurrence	10	19.6
Recurrence		
Platinum sensitive	26	51.0
Platinum resistance	12	23.5
Lost to follow up	3	5.9
TIL % score		
<20	21	41.2
20–40	16	31.4
>40	10	19.6
Not available	4	7.8
HRD mutation		
Positive		
* BRCA1/2*	18	35.3
Others	4	7.8
Negative	27	52.9
Not available	2	3.9

## Data Availability

The data that support the finding of this study are available from the corresponding author, upon reasonable request.

## Supplementary Materials

The following supporting information can be downloaded at: https://www.mdpi.com/article/10.3390/genes14040785/s1, Figure S1. The survival analysis for 4 differentially expressed genes in our study and TCGA high-grade serous ovarian carcinoma data; Figure S2: The expression pattern of the TCR repertoire in patient groups divided based on HRD mutation.
