# Supplementary material for: T-Cell Receptor Repertoire Characteristics Associated with Prognostic Significance in High-Grade Serous Ovarian Carcinoma
Source: Genes (Basel). 2023 Mar 24;14(4):785. doi: 10.3390/genes14040785 (PMC10137910; doi:10.3390/genes14040785)
Supplement: Supplementary file 1 [file genes-14-00785-s001.zip › genes-2250774-supplementary.pdf]

genes

MDPI

Article

T-Cell Receptor Repertoire Characteristics Associated with Prognostic Significance in High-Grade Serous Ovarian Carcinoma

Ju-Won Kim [1], Sewha Kim [2,3], So-Yun Yang [1], Je-Gun Joung [1,4,5,*] and Sohyun Hwang [1,2,5,*]

1 Department of Biomedical Science, College of Life Science, CHA University, Seongnam 13488, Republic of Korea; seven1009@naver.com (J.-W.K.)
2 Department of Pathology, CHA Bundang Medical Center, CHA University School of Medicine, Seongnam 13496, Republic of Korea
3 Department of Pathology, Korea Medical Institute, Seoul 03173, Republic of Korea
4 Institute for Biomedical Informatics, CHA University School of Medicine, Seongnam 13488, Republic of Korea
5 CHA Future Medicine Research Institute, CHA Bundang Medical Center, Seongnam 13488, Republic of Korea
* Correspondence: jgjoung@cha.ac.kr (J.-G.J.); blissfulwin@cha.ac.kr (S.H.)

Supplementary Figures:

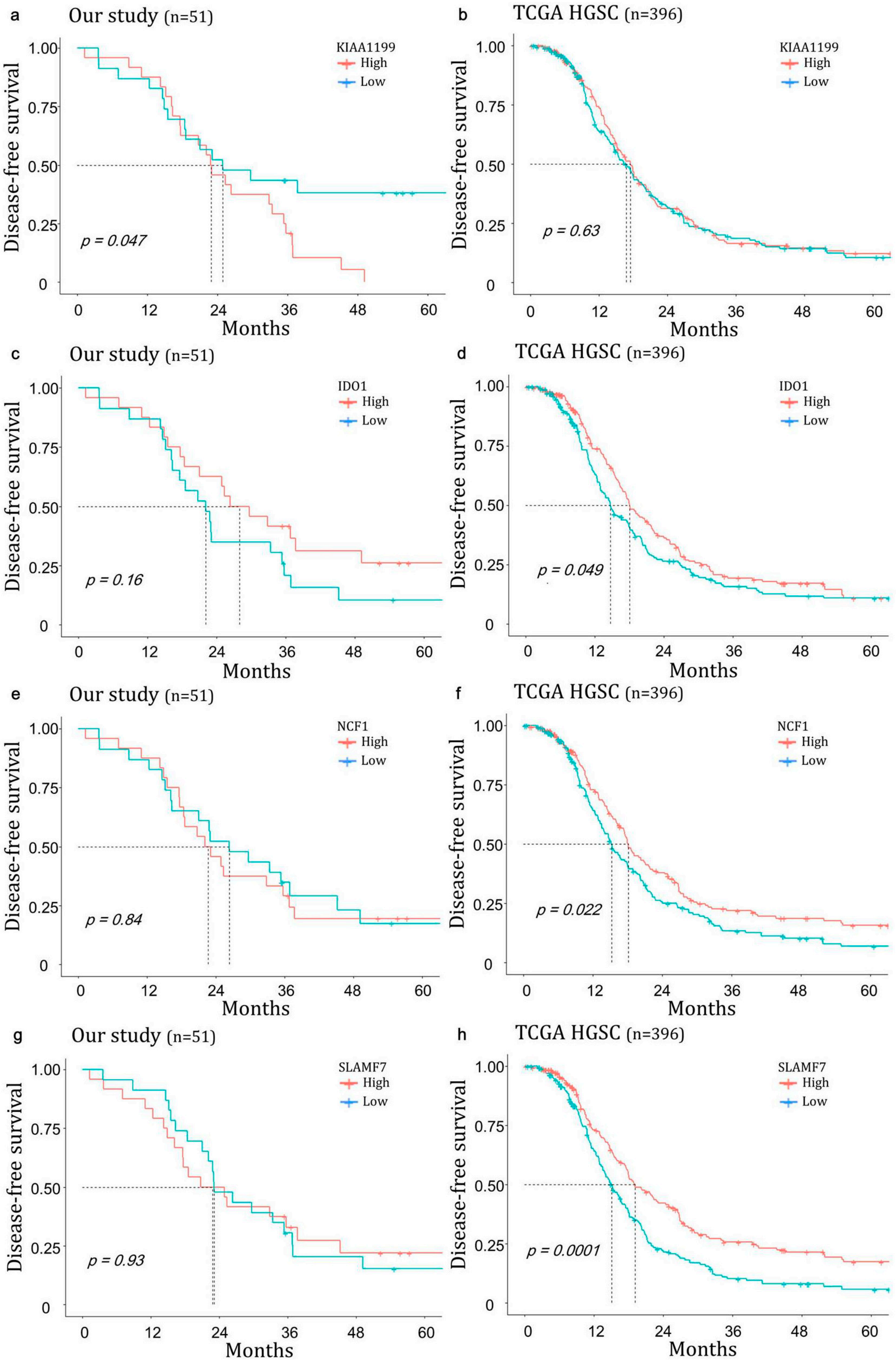

Figure S1. The survival analysis for 4 differentially expressed genes in our study and TCGA high-grade serous ovarian carcinoma (HGSC) data. For survival analysis, disease-free survival data were analyzed in both data. Survival results were shown for *KIAA1199* in our study (a) and TCGA HGSC (b), for *IDO1* in our study (c) and TCGA HGSC (d) for *NCF1* in our study (e) and TCGA HGSC (f) and for *SLAMF7* in our study (g) and TCGA HGSC data (f), respectively.

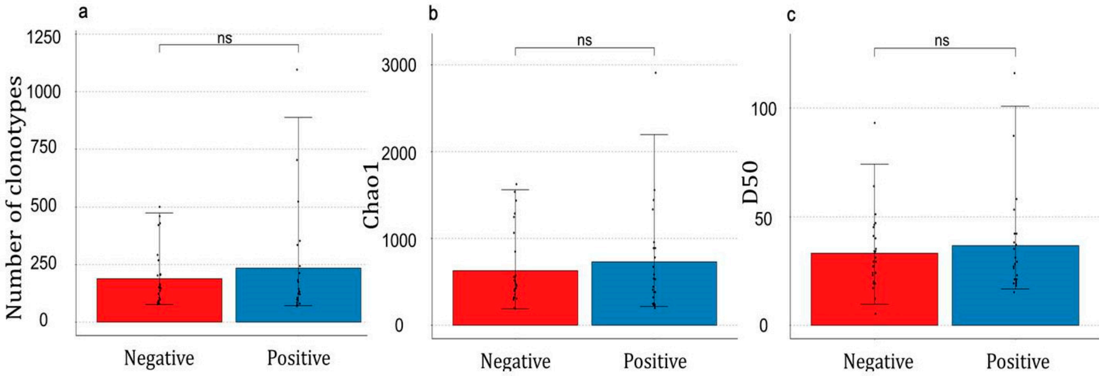

Figure S2. The expression pattern of the TCR repertoire in patient groups divided based on HRD mutation. **(a)** T-cell receptor counts of unique TCR clonotypes. **(b–c)** Richness and diversity of TCR estimated by Chao1, and D50 index in patient groups divided based on HRD mutation. **(a–c)** Non-significant *p*-values are indicated as ns.
